# Cadmium Toxicity to *Zea mays* and Its Implications for the Uptake of Other Heavy Metals by the Plant

**DOI:** 10.3390/molecules31081317

**Published:** 2026-04-17

**Authors:** Jadwiga Wyszkowska, Agata Borowik, Magdalena Zaborowska, Jan Kucharski

**Affiliations:** Department of Soil Science and Microbiology, Faculty of Agriculture and Forestry, University of Warmia and Mazury in Olsztyn, Plac Łódzki 3, 10-727 Olsztyn, Poland; agata.borowik@uwm.edu.pl (A.B.); m.zaborowska@uwm.edu.pl (M.Z.); jan.kucharski@uwm.edu.pl (J.K.)

**Keywords:** maize yield, toxicity index, translocation index, heavy metal uptake, organic sorbents

## Abstract

Cadmium is an element that is unnecessary for the functioning of plant and animal organisms, and its widespread presence in the environment poses a serious threat to human and animal health. Therefore, effective methods are being sought to remediate soils contaminated with this element, including through the enrichment of degraded soils with organic matter. To this end, the effectiveness of selected organic sorbents, including starch, fermented bark, compost and humic acids, in mitigating the transfer of cadmium and other heavy metals from soil to plants was assessed. Model studies compared the effects of 15 and 30 mg of cadmium (Cd) per kg of soil with an uncontaminated control sample. The sorbents were applied on a carbon basis at a rate of 3 g C per kg of soil. The test plant was *Zea mays*. Cadmium was found to significantly impair plant growth, causing reductions of 21%, 85%, and 77% in leaf greenness, aboveground biomass and root biomass, respectively. Excess cadmium increased the translocation of lead, chromium, copper, nickel, zinc, iron, and manganese from the roots to the aboveground parts of the plant, while simultaneously limiting their uptake. All of the organic sorbents tested reduced the negative impact of cadmium on leaf greenness, except starch. Compost and HumiAgra significantly improved the condition of *Zea mays* plants weakened by cadmium exposure. Cadmium contamination increased soil acidification. pH was positively correlated with maize yield and the SPAD leaf greenness index and negatively correlated with the cadmium translocation index and cadmium content in the aboveground parts of maize. Compost and humic acids are among the most effective and practically feasible approaches for reducing cadmium bioavailability in soil and its accumulation in *Zea mays*, and are therefore recommended for the remediation of cadmium-contaminated soils.

## 1. Introduction

Cadmium (Cd), due to its high toxicity [1,2], persistence, non-biodegradability, and bioavailability to organisms at all trophic levels [3,4], poses one of the most serious environmental challenges [5,6,7]. Although cadmium is not an essential element for plant growth, it is readily taken up by root systems and transported to other parts of the plant [8,9], resulting in its accumulation in edible tissues and posing a significant risk to food safety and public health [10,11]. The ease with which cadmium is taken up by the plant root system stems from the structural similarity of Cd^2+^ ions to phosphorus and zinc, as well as the use of the same membrane transporters responsible for the uptake of macro- and micronutrients from the soil [9]. Once inside root cells, Cd interacts with the sulfhydryl groups (–SH) of proteins, leading to disruptions in protein structure and thereby destabilizing metabolic processes and enzymatic functions. Cadmium induces oxidative stress. Excessive accumulation of reactive oxygen species (ROS) leads to damage to proteins, pigments, and nucleic acids, which disrupts cell integrity and promotes further cadmium movement within the plant [12]. Consequently, cadmium causes complex physiological disorders in plants, primarily manifested as growth inhibition, morphological abnormalities, inhibition of photosynthesis, and oxidative damage [13].

A plant’s sensitivity to cadmium is also determined by how it interferes with the soil’s physicochemical properties. Its sorption in soils is mediated by complexation via van der Waals forces, electrostatic interactions, and chemical interactions [14]. Cadmium tends to accumulate at the organic-mineral phase boundaries within soil aggregates, where its retention is determined by the composition and structural complexity of organic matter, as well as its interactions with Fe and Al oxides [15]. These interferences promote metal accumulation in fine soil fractions, potentially affecting aggregate stability and structural properties. The sorption capacity for cadmium is moderated by pH, cation exchange capacity (CEC), and soil organic matter (SOM) content. Therefore, the formation of complexes with soil organic matter, through interaction with functional groups, influences its reactivity and sorption capacities [14]. An increase in pH leads to higher cadmium sorption capacity, which in turn moderates soil solution dynamics and ion transport. Importantly, cadmium, as the Cd^2+^ cation, competes with cations such as Ca^2+^, Mg^2+^, and Zn^2+^ for binding sites in the sorption complex, determined by cation exchange capacity (CEC), significantly interfering with nutrient availability [16]. The negative impact of metal on soil health also stems from its toxicity to soil microorganisms. This toxicity results in oxidative stress, which leads to bacterial cell lysis [17].

The Agency for Toxic Substances and Disease Registry has classified cadmium as one of 275 substances identified as priorities [18]. Even low-dose, long-term exposure to cadmium can lead to health conditions such as osteoporosis and cardiovascular damage, as well as increasing the risk of cancers of the breast, lung, prostate, nasopharynx, pancreas and kidney [10,19,20]. Its biological half-life in the human body ranges from 16 to 30 years [21]. This is particularly alarming in light of data reported by the World Health Organization (WHO), indicating that soil contamination contributes to over 0.5 million premature deaths worldwide each year [22].

In the 20th century, the Cd content in soil increased by 50% [23]. Currently, the average content of Cd in the topsoil layer worldwide is 0.36 mg kg^−1^ dry weight [23], and in soils in the European Union, it is 0.20 mg kg^−1^ [24]. However, cadmium content in soils exhibits significant spatial variability; for example, it is 6.3 mg kg^−1^ in Nigerian soils, 5.0 mg kg^−1^ in Malaysian soils, and 2.5 mg kg^−1^ in Ecuadorian soils [25]. In Poland, the average Cd content in arable soils increased from 0.7 mg kg^−1^ in 1995 to 3.4 mg kg^−1^ in 2020 over the past 25 years [26]. De Vries et al. [27] point out that the Mediterranean region is particularly susceptible to cadmium accumulation due to local soil properties that influence metal retention. Mapping of European soils, based on an analysis of 21,682 samples, revealed that cadmium concentrations in the topsoil range from 0.07 mg kg^−1^ to 65.4 mg kg^−1^ [24].The highest Cd concentrations are found in industrial soils, followed by roadside and agricultural soils [6,25]. Zulfiqar et al. [9] emphasize that the threshold level for cadmium in agricultural soils is approximately 100 mg kg^–1^. The main anthropogenic sources of this metal’s emissions are the metallurgical industry, including the production and refining of non-ferrous metals, as well as the production of coloring pigments and Ni-Cd batteries [28], fossil fuel combustion, transportation, and the use of phosphate fertilizers, ash, and sewage sludge [25].

According to the European Environment Agency, there are currently approximately 2.8 million sites in Europe [29] classified as potentially contaminated, and about 14% of these are likely to require remediation. Globally, between 0.9 and 1.4 billion people live in areas characterized by high risks to public health and the environment [30]. Given the increasing extent of cadmium soil contamination [3,31,32], and its well-documented consequences for human health [10,33,34,35], and ecosystem functioning [36,37], there is an urgent need to develop remediation methods [38,39,40], that combine high effectiveness with economic viability and environmental safety [31,41]. In line with the objectives of the European Union soil protection strategy [42], which aims to reduce soil contamination to levels that no longer pose a risk to human health by 2050, there is a need to develop remediation methods that are effective, economical, and environmentally friendly [5,39,43]. Among these methods, organic sorbents deserve particular attention [2,44,45,46,47] due to their ability to immobilize heavy metals via numerous functional groups, while simultaneously improving soil properties and promoting plant growth and development [7,38].

There are many reasons to justify the choice of sorbents. The use of starch is based on two main reasons. First, it is the most abundant polysaccharide in nature, and its ability to interact with contaminants of varying hydrophilicity arises from its glycosidic structure, which contains hydroxyl groups [48]. Second, the starch market was estimated at USD 13.1 million, with production in the European Union totaling 9.2 million tons [49,50]. Similar considerations guided the selection of prefermented bark as a cadmium adsorbent. According to Junior et al. [51], *Pinus elliottii* bark can adsorb cadmium at a rate of 90%. From an economic perspective, approximately 200 million m^3^ of bark were produced as waste in 2021, whose disposal poses both logistical and environmental challenges [52]. Compost, as a lignocellulosic resource and a source of reducing agents, acts as a sorbent that immobilizes cadmium in the soil, thereby limiting its bioavailability [53]. Additionally, due to the observed decline in C_org_ content in arable soils, its use is carefully considered [54]. Humic acids present in the soil play a key role in metal sequestration through their functional groups, which facilitate the formation of humates. Their efficacy against cadmium reaches 96.18% [55,56].

This study’s innovative approach lies in its use of four structurally distinct sorbents, ranging from simple polysaccharides to highly humified fractions. These sorbents are readily available, cost-effective and biodegradable. This selection of sorbents enables the analysis of diverse cadmium binding mechanisms and the evaluation of their effectiveness in limiting cadmium mobility, and aligns closely with the “4 per 1000: Soils for Food Security and Climate” initiative. This initiative promotes the vision of soils enriched in soil organic carbon (SOC), aiming to achieve a positive impact on climate change by 2050 [57]. The growing threat posed by cadmium accumulation in agricultural soils [3,25,32] and in degraded areas [26,46,58] made it imperative to conduct this experiment. Therefore, the primary objective of the study was to comprehensively determine the effectiveness of selected organic sorbents: starch, fermented bark, compost, and humic acids in reducing the mobility of cadmium and other heavy metals in the Cd-contaminated soil–plant system. The study also focused on identifying the most effective sorbents that can be recommended in practice as cost-effective and environmentally safe tools to support the remediation of degraded sites. The following research hypothesis was formulated: the use of organic sorbents leads to a significant reduction in cadmium uptake by *Zea mays*, thereby limiting the negative effects of cadmium on plant growth and development, as well as reducing the accumulation of heavy metals in plant tissues.

## 2. Results

### 2.1. Cadmium Toxicity to Zea mays

**Figure 1:**
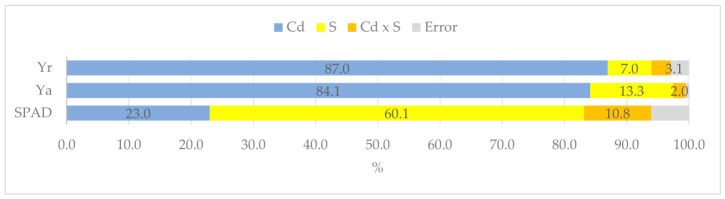
The extent of the influence of cadmium (Cd) and organic sorbents (S) on the greenness index (SPAD), aboveground biomass (Ya), and root biomass (Yr), as measured by η^2^.

**Figure 2:**
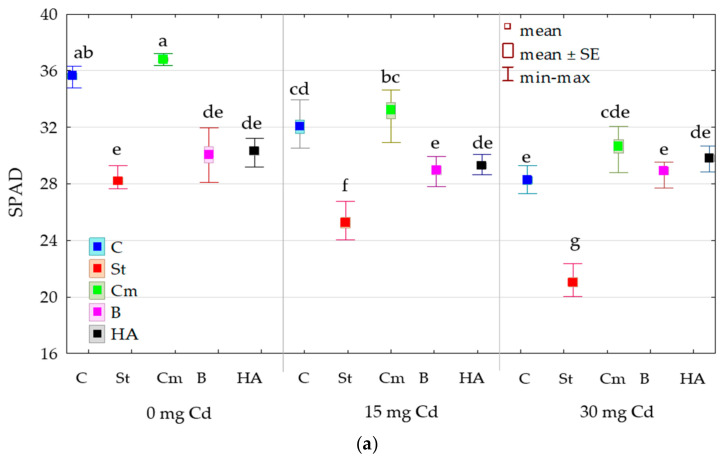

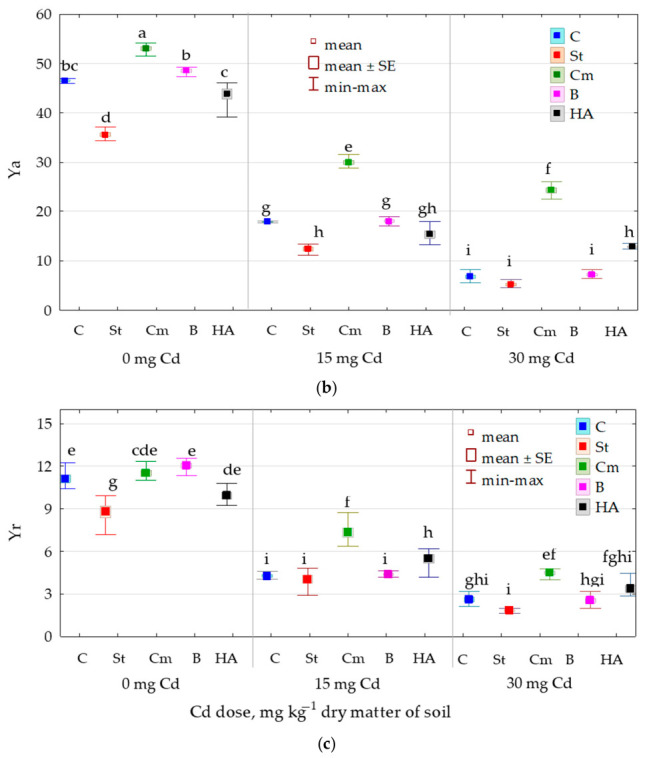
The effect of cadmium and sorbents on (**a**) leaf greenness index (SPAD); (**b**) d.m. yield of aboveground parts (Ya) in g pot^−1^; (**c**) d.m. yield of roots (Yr) in g pot^−1^ of *Zea mays*. Explanations: C—control, St—starch, B—bark, Cm—compost, and HA—HumiAgra; lowercase letters indicate the significance of differences.

Both cadmium contamination of the soil and the application of organic matter to the soil influenced the maize leaf greenness index (SPAD) (Figure 1). Cadmium accounted for 84% of the above-ground yield and 87% of the root yield. In the case of the SPAD index, the type of sorbent (60%) had a stronger influence than the cadmium dose (23%). Cadmium applied to the soil at rates of 15 and 30 mg Cd kg^−1^ reduced the SPAD value from an average of 32.189 to 29.737 (15 mg Cd dose) and 27.726 (30 mg Cd dose) (Figure 2). In contrast, the tested organic materials had varying effects. Compost increased the SPAD value from an average of 31.960 to 33.535, whereas the other substances decreased it. Starch had the greatest impact, reducing the SPAD value to as low as 24.827. Naturally, the differences in leaf greenness had repercussions on the growth and development of *Zea mays*, and consequently impacted the plant’s yield. The yield of maize decreased as cadmium contamination in the soil increased. Under the influence of 30 mg Cd kg^−1^ of soil, the yield of aboveground parts decreased 6.7-fold in the control treatment, and that of roots decreased 4.3-fold. The addition of starch and bark to the soil did not mitigate the negative effects on either the aboveground or root parts. Compost effectively mitigated the toxic effects of cadmium on the yield of aboveground plant parts. This parameter was reduced only 2.2-fold, and root biomass 2.6-fold, compared to the control. HumiAgra had a similar effect to compost, as under its influence, the reduction in above-ground yield decreased from 6.7-fold to 3.4-fold, and in roots from 4.3-fold to 3.0-fold.

**Figure 3:**
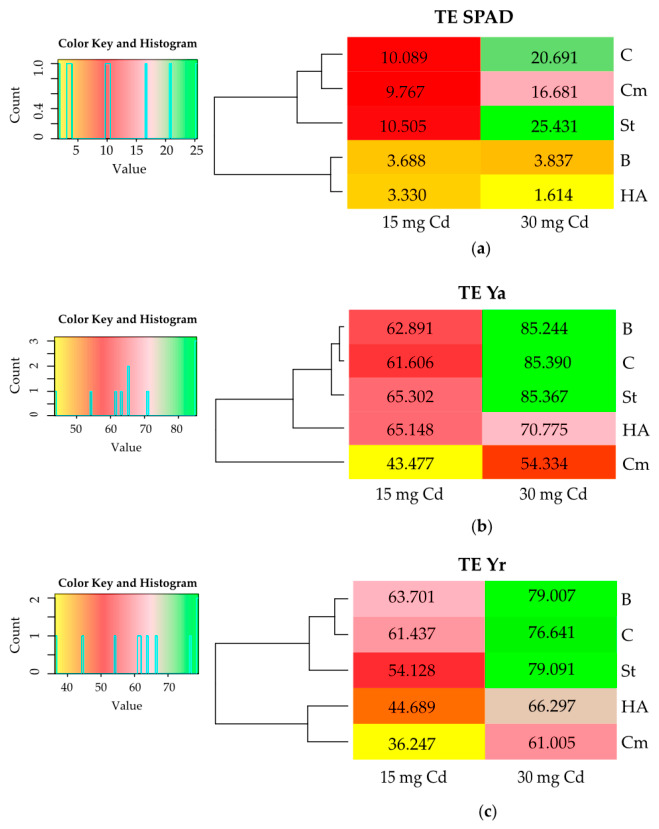
Cadmium toxicity index (TE) for *Zea mays* calculated based on (**a**) leaf greenness index (SPAD); (**b**) dry matter yield of aboveground parts (Ya); (**c**) dry matter yield of roots (Yr). Explanations: C—control, St—starch, B—bark, Cm—compost, and HA—HumiAgra.

The cadmium toxicity index (TE) for *Zea mays* depended on the amount of this element introduced into the soil (Figure 3). The average index resulting from the lower Cd dose (15 mg kg^−1^) was 7.467 for SPAD, 59.685 for aboveground parts, and 52.040 for roots, while the higher Cd dose (30 mg kg^−1^) resulted in values of 13.651, 76.222, and 72.408 for SPAD, aboveground parts, and roots, respectively. The magnitude of the TE indices indicates high cadmium toxicity for maize. None of the applied organic amendments reversed the adverse effects of cadmium on the relationship between maize and soil Cd levels. However, the toxic impact of the metal on plants grown in soils containing 15 or 30 mg kg^−1^ Cd was significantly reduced by compost. In contrast, HumiAgra only mitigated the toxic effects of cadmium on maize grown in soil containing 30 mg of cadmium.

### 2.2. Cadmium and Other Heavy Metal Content and Translocation in Zea mays

**Figure 4:**
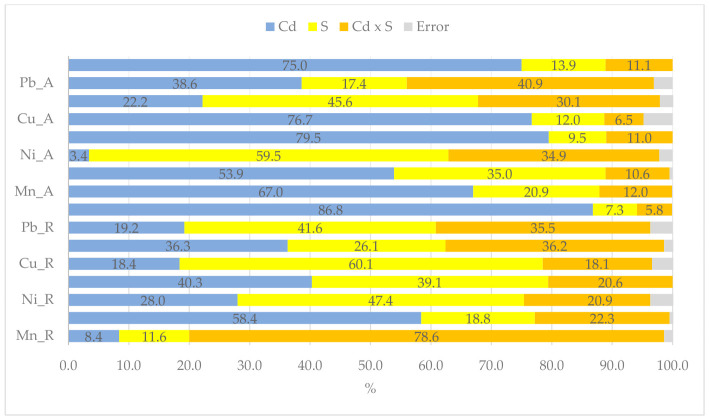
The extent of the influence of cadmium (Cd) and organic sorbents (S) on heavy metals content in aboveground parts (A) and roots (R), as measured by η^2^.

As with the yield of *Zea mays*, the concentration of individual elements in the above-ground parts and roots was affected by the cadmium added to the soil and the sorbents used (Figure 4). Both factors affected the content of individual metallic elements to varying degrees. In the aboveground parts, the cadmium dose most strongly determined the zinc (79%), copper (77%), cadmium (75%), manganese (67%), and iron (54%) content. In the aboveground plant parts, cadmium dose had the greatest influence on the content of zinc (80%), copper (76%), cadmium (75%), manganese (67%), and iron (54%). In the roots, cadmium exposure most strongly affected cadmium content (87%), followed by iron (58%), zinc (40%), and chromium (35%). Sorbents had the greatest effect on the nickel (59%), chromium (46%), and iron (35%) content in the aboveground parts and the copper (60%), nickel (47%), lead (42%), and zinc (39%) content in the roots.

The increased cadmium concentration in the soil not only led to higher Cd levels in the aboveground parts of maize (Table A1, Appendix A) but also disrupted the ionic balance of other elements. In control plants without organic sorbents, the content of copper, zinc, iron and manganese increased in parallel with cadmium, while chromium content decreased. In contrast, cadmium had a varied effect on the accumulation of lead and nickel in the aboveground parts of maize. A lower dose (15 mg) caused an increase in the accumulation of these elements, while a higher dose (30 mg) caused a decrease.

The sorbents also modified the concentration of the studied elements in the aboveground parts of *Zea mays*. In contrast, cadmium content increased, while nickel and iron content decreased in plants grown on contaminated soil, irrespective of the contamination level. The effect of the sorbents on the content of the other elements varied depending not only on the type of sorbent, but also on the level of cadmium contamination in the soil. Starch, compost, bark, and HumiAgra reduced the lead and copper content in the aboveground parts of maize grown in soil with 15 mg Cd kg^−1^ added. All of the aforementioned sorbents, with the exception of bark, had a similar effect on chromium content.

In the roots of *Zea mays* grown in soil without sorbents, cadmium content increased with the degree of soil contamination by this element (Table A2). A synergistic effect was also observed between cadmium and copper content, whereas an antagonistic effect was observed between cadmium and lead, chromium, zinc, nickel, iron and manganese content. All of the sorbents reduced the content of lead, chromium, zinc, and nickel in the roots of *Zea mays* grown in cadmium-uncontaminated soil. Cadmium content in the roots decreased following soil supplementation with compost, whereas it increased after the application of bark and HumiAgra. Adding starch to the soil increased the copper content in the roots, while applying compost reduced it. Bark and HumiAgra did not affect copper accumulation in the roots. Conversely, starch and compost reduced iron content, while bark and HumiAgra increased its accumulation. Starch, compost, and HumiAgra reduced manganese content in the roots. Bark had no effect on this parameter. All sorbents reduced cadmium content in the roots of plants grown in soil contaminated with 15 mg Cd, as well as in soil with higher contamination (30 mg Cd). In the latter case, starch had a different effect. The addition of bark and HumiAgra to cadmium-contaminated soil significantly reduced the levels of lead, zinc, nickel and manganese in the roots.

Similar to the sorbents mentioned above, compost also affected nickel levels. In the roots of *Zea mays* grown in soil more heavily contaminated with Cd (30 mg kg^−1^), all tested sorbents significantly reduced the concentrations of zinc and nickel, and, except for starch, also those of cadmium, lead, and copper.

**Figure 5:**
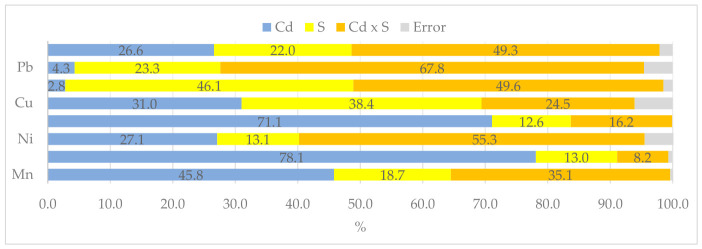
The extent of the influence of cadmium (Cd) and organic sorbents (S) on the translocation factor (TF) of heavy metals, as measured by η^2^.

The data presented in Figure 5 clearly demonstrate that both cadmium added to the soil and organic sorbents influenced the translocation factor (TF). Cadmium most strongly determined the translocation of iron (78%), zinc (71%), manganese (46%), copper (31%), and cadmium (27%), while sorbents influenced the translocation of chromium (46%), copper (38%), lead (23%) and cadmium (22%), zinc, nickel, and iron (13%), and manganese (19%).

**Figure 6:**
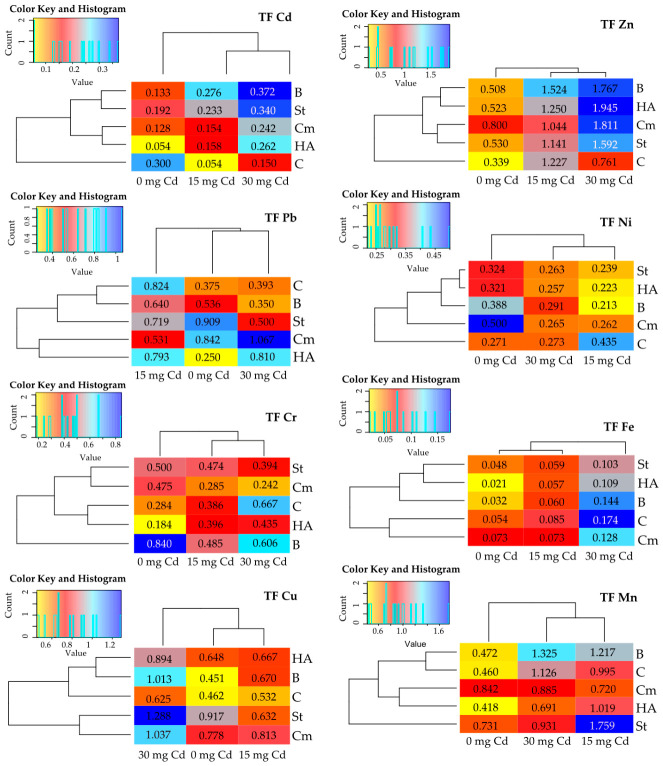
The effect of cadmium and sorbents on the translocation Index (TF) of heavy metals in *Zea mays*. Explanations: C—control, St—starch, B—bark, Cm—compost, HA—HumiAgra.

The concentration of cadmium and other heavy metals in specific plant organs was reflected in their translocation factor (TF) (Figure 6). In cadmium-contaminated soils, the TF index reached its highest values for zinc, manganese, copper, and lead. The presence of excess cadmium in the soil increased the mobility of lead, chromium, copper, nickel, zinc, iron, and manganese from the roots to the aboveground parts, while it decreased the mobility of cadmium. The addition of all organic sorbents to uncontaminated soil significantly reduced the transport of cadmium and iron from roots to aboveground parts (with the exception of compost). All sorbents enhanced the transport of zinc and nickel. Starch and compost further enhanced the transport of lead, chromium, and manganese, whereas starch, compost, and HumiAgra promoted copper transport. Compost alone enhanced the transport of iron. The application of sorbents to cadmium-contaminated soil at both levels increased the translocation of cadmium and copper from the roots to the aboveground parts of *Zea mays*. The opposite trend was observed for iron and nickel, with the exception of bark applied to soil containing 30 mg kg^−1^ Cd.

### 2.3. Uptake of Cadmium and Other Heavy Metals by Zea mays

**Figure 7:**
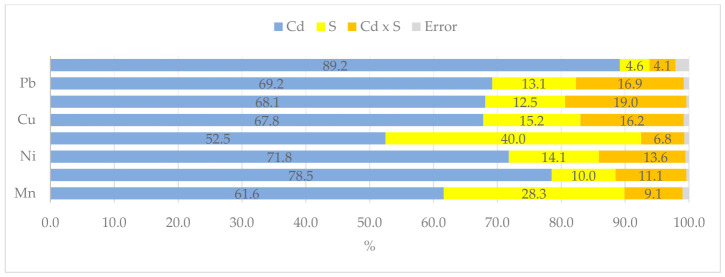
The extent of the influence of cadmium (Cd) and organic sorbents (S) on heavy metals uptake by *Zea mays*, as measured by η^2^.

The uptake of metallic elements from the soil was inhibited by excess cadmium in the soil, as confirmed by the eta-squared values (Figure 7). These values indicate that the uptake of elements ranged from 52% (Zn) to 89% (Cd) depending on the concentration of cadmium in the soil, and from 5% (Cd) to 40% (Zn) depending on soil supplementation with sorbents.

*Zea mays* grown in cadmium-contaminated soil, but without the addition of sorbents, showed higher uptake of cadmium from the soil, while the uptake of lead, chromium, copper, zinc, nickel, iron, and manganese was lower (Table 1). The effect of the sorbents used on the uptake of the tested metals by maize grown in uncontaminated soil was inconclusive. Starch and HumiAgra reduced the uptake of Cd, Pb, Cr, Cu, Zn, Ni, Fe, and Mn by *Zea mays*; compost reduced the uptake of Cd, Pb, Cr, Cu, Ni, and Fe; and bark reduced the uptake of Cd, Pb, Cu, and Ni. In contrast, compost increased the uptake of Zn and Mn, while bark increased the uptake of Cr, Zn, Fe, and Mn. The effect of the tested sorbents on the uptake of metals by *Zea mays* in cadmium-contaminated soil was unclear, although the plants accumulated more cadmium, particularly in plots with 30 mg Cd. Among all the sorbents, compost and HumiAgra deserve special attention, as they promoted increased uptake of all the analyzed elements. Conversely, starch significantly impaired the uptake of Cr, Cu, Zn, Ni, Fe, and Mn, while bark impaired the uptake of Pb and Fe.

### 2.4. Effects of Cadmium and Sorbents on Soil pH and the Content of Carbon and Nitrogen

**Figure 8:**
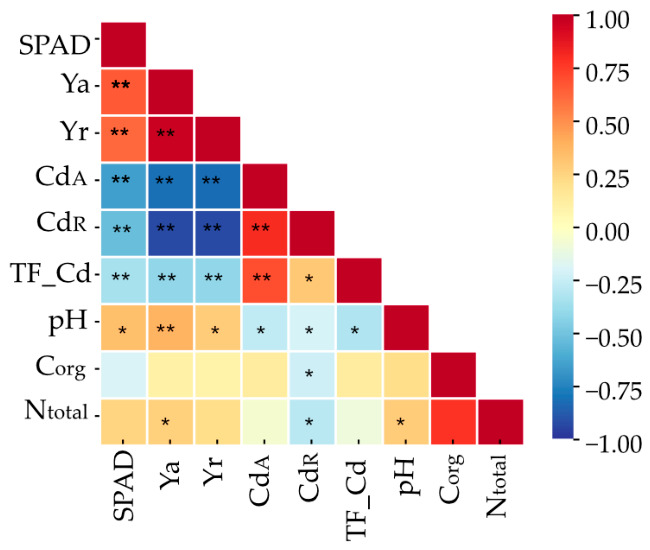
Correlation between soil pH, organic carbon content (C_org_), total nitrogen (N_total_), and the most important parameters obtained in the study. (n = 60, * *p* > 0.05, ** *p* > 0.01). SPAD—leaf greenness index of *Zea mays*, Ya—aboveground yield, Yr—root yield, TF_Cd—cadmium translocation index, Cd_A—cadmium content in aboveground parts, Cd_R—cadmium content in roots.

Cadmium contamination significantly reduced soil pH (Figure 8). The mean values, regardless of the type of sorbent applied, showed that the highest pH was recorded in uncontaminated soil (4.521), significantly lower in soil contaminated with 15 mg Cd kg^−1^ (4.457), and lowest in soil contaminated with 30 mg Cd kg^−1^ (4.467). Similarly, mean values independent of cadmium levels indicated that sorbents also had a significant effect on soil pH. Compost (pH 4.692) and starch (pH 4.505) had slightly alkalizing effects, whereas fermented bark lowered soil pH (4.288).

Application of all organic sorbents to both uncontaminated and cadmium-contaminated soils resulted in a significant increase in organic carbon (C_org_) and total nitrogen (Nt_otal_), while cadmium itself had no statistically significant effect on these parameters. In the control soil (without Cd addition), these amendments increased C_org_ from 22.768% (bark) to 28.413% (compost) and N_total_ from 8.157% (starch) to 32.570% (compost). Similar trends were observed in soils exposed to cadmium stress.

In conclusion, the tested sorbents can be ranked for their effectiveness in increasing C_org_, regardless of cadmium application, as follows: compost (27.483%) > starch (24.714%) > HumiAgra (24.117%) > bark (22.752%). For N_total_, their effectiveness was ranked as: compost (32.544%) > HumiAgra (27.059%) > bark (22.104%) > starch (10.312%).

Summarizing the data in Table A3, it can be observed that the C_org_ content was significantly positively correlated with N_total_ content (Figure 8). However, C_org_ accumulation in the soil did not exhibit any significant correlation with the other examined characteristics. Furthermore, the N_total_ content in the soil was positively correlated with aboveground yield and soil pH, and negatively correlated with cadmium content in maize roots. Soil pH was positively correlated with maize yield and the SPAD leaf greenness index, and negatively correlated with the cadmium translocation factor and cadmium content in the aboveground parts of maize.

### 2.5. Interactions Between Zea mays Yield and the Uptake and Translocation of Metal Elements

**Figure 9:**
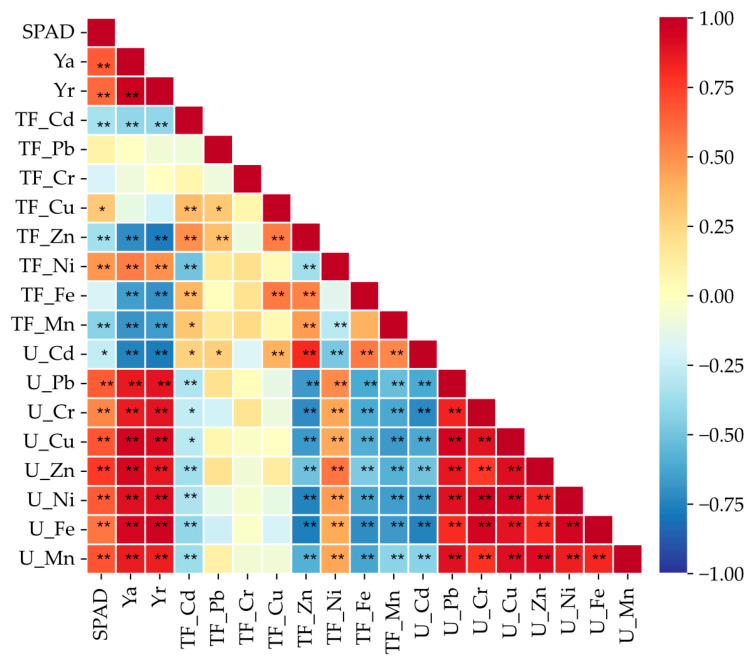
Correlation between the leaf greenness index of *Zea mays* (SPAD), aboveground yield (Ya), root yield (Yr), heavy metal translocation index (TF), and heavy metals uptake (U) (*n* = 60, * *p* > 0.05, ** *p* > 0.01).

**Figure 10:**
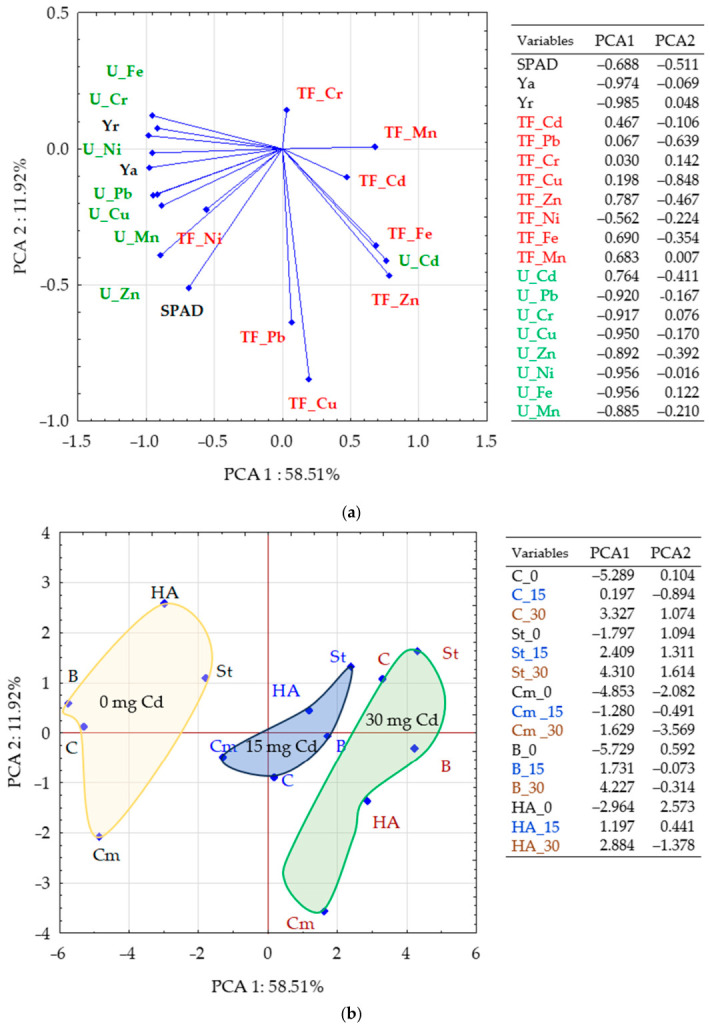
Principal Component Analysis (PCA): (**a**) a plot showing the lengths and directions of vectors indicating the strength of the variables’ influence on the data structure; (**b**) the distribution of the cases studied in the space of the first two principal components (PC1 and PC2). Explanations: C—control, St—starch, B—bark, Cm—compost, HA—HumiAgra.

The greenness index (SPAD) and the yield of the aboveground parts and roots of *Zea mays* were significantly negatively correlated with the translocation of Cd, Zn, and Mn in the plant. They were also positively correlated with the translocation of Ni. The analyzed parameter was not correlated with Cr and Pb (Figure 9 and Figure 10). The Cd translocation index was significantly negatively correlated with the uptake of all elements except Cd itself, as well as with Ni translocation. It was also positively correlated with the translocation of Cu, Zn, and Fe. Cd uptake was negatively correlated with the uptake of all elements, whereas the uptake of the remaining elements was significantly positively correlated with each other.

The distances of the effects of organic sorbents, depending on the level of soil cadmium contamination, demonstrate their significant influence. Simultaneously, they reveal differences in their effects on maize yield, the leaf greenness index, and the translocation and uptake of trace elements. The effects of sorbents on the studied parameters can be classified into three categories, depending on the level of cadmium contamination in the soil (Figure 10). Cadmium applied to the soil at a concentration of 30 mg Cd had the most adverse effect on plant condition, while that applied at 15 mg had a significantly lesser effect. Among the tested sorbents, compost added to the soil at the lower contamination level was the most effective in mitigating the negative impact of cadmium on plants.

## 3. Discussion

In the present study, applying 15 mg Cd kg^−1^ soil resulted in a 61.6% reduction in aboveground biomass and a 61.4% reduction in root biomass. Increasing the cadmium dose to 30 mg kg^−1^ of soil intensified these negative effects, resulting in a reduction in aboveground and root biomass by 85.4% and 76.6%, respectively. It was also noted that a significant reduction in the leaf greenness index (SPAD) of maize, by 10.1% under the influence of 15 mg Cd kg^−1^ of soil and by 20.7% under the influence of 30 mg Cd kg^−1^ of soil, indicates disturbances in the photosynthesis process. This is consistent with the results of Obadoni et al. [59], who demonstrated a reduction in chlorophyll content in the leaves of *Zea mays* grown in soil contaminated with cadmium at levels of 5–30 mg Cd kg^−1^. Reduced photosynthetic efficiency also leads to stunted plant growth and development, which is a consequence of reduced water and nutrient uptake [21,60,61].

The disruption to the growth and development of *Zea mays* observed in this study when grown in cadmium-contaminated soil is consistent with recent reports [13,62,63]This results in both a reduction in its biomass and an interference with the process of photosynthesis. Disturbances to maize growth and development result in a reduction in its biomass [45,50].

Similarly, Elik and Gül [64] and Kavian [65] reported that excessive cadmium content in the soil leads to significant reductions in shoot length and disturbances in root system development, consequently decreasing the biomass of *Zea mays*. Growth and developmental disturbances in *Zea mays* grown in cadmium-contaminated soil result from both the direct effects of Cd and the indirect oxidative stress (ROS) induced by this metal [12,64]. It is important to emphasize the multifaceted and complex nature of this process. Excess reactive oxygen species (ROS) can initiate lipid peroxidation, oxidative protein modifications, mitochondrial membrane depolarization, genetic material damage, and activation of the programmed cell death pathway. These processes lead to disturbances in oxidative phosphorylation and a reduction in ATP synthesis efficiency [66,67]. Cd^2+^ ions are mainly taken up by plants through passive transport [68] and via non-selective metal transporters, such as the iron-regulated transporter 1 (IRT1), the ZRT/IRT-like proteins (ZIPs), and the natural resistance-associated macro-phage proteins (NRAMPs), which are naturally responsible for the transport of Fe^2+^, Zn^2+^, Mn^2+^, and other ions [67,69]. Cadmium can also enter through calcium channels, competing with Ca^2+^ for transport sites, thereby increasing its accumulation in roots [13]. Once inside cells, Cd^2+^ is rapidly bound by phytochelatins and metallothioneins, which reduces its toxicity but does not necessarily prevent its movement to aboveground parts [70]. Another phenomenon that should be addressed is the variation in the accumulation of Cd^2+^ ions among individual plant organs. This represents one of the key factors determining their adaptive capacity to grow under stress conditions induced by excess cadmium in the soil [13,71]. After being taken up by the roots, a significant proportion of Cd^2+^ becomes localized in the cell wall or vacuoles, while another proportion is transported to the xylem for further translocation within the plant [72]. It may also be distributed to the phloem [73].

In our own studies, cadmium negatively affected the yield of maize and altered the plant’s chemical composition. An increase in the translocation of other metals from the roots to the aboveground parts was observed. In the aboveground parts, the contents of cadmium, copper, zinc, iron, and manganese increased, whereas the chromium content decreased. An increase in cadmium content was also observed in the roots as soil contamination increased, but, in contrast to the aboveground parts, a decrease in the content of lead, chromium, zinc, nickel, iron and manganese was found. The results of our research, as well as data from other researchers [57,61,64] suggest that elevated cadmium levels in the soil may intensify the transport of other metals to the stems and leaves of plants. These changes result from disruptions to their uptake and translocation caused by excess cadmium [71,74,75].

Hédiji et al. [76] report that cadmium (Cd) reduces the Zn, Cu and Mn content in the flowers of *Lycopersicon esculentum*, while increasing the Fe content. Significant modulation of metal transfer from soil to plants and their accumulation was demonstrated in *Ocimum basilicum* L. by Lykas et al. [77], in *Melissa officinalis* by Adamczyk-Szabela et al. [78], in *Avena sativa* L. and *Sinapis alba* L. by [46], in *Zea mays* L. by Yue et al. [79], Elik and Gül [64], and by Borowik et al. [80].

Changes in the translocation of multiple heavy metals in the presence of excess Cd provide evidence of competitive and synergistic interactions between metals at the level of the ion transport pathway [81]. This phenomenon should be considered highly detrimental due to the risk of disrupting the food chain in humans and animals.

The ability of plants to translocate metals can be assessed using the translocation index (TF) [79,82]. Huang et al. [83] emphasize that average heavy metal translocation rates are higher in historically contaminated soils than in recently contaminated soils. In our own studies, cadmium contamination of the soil was relatively short (55 days). It increased the translocation of Pb, Cr, Cu, Ni, Zn, Fe, and Mn from the roots to the aboveground parts of *Zea mays*, while simultaneously reducing the mobility of cadmium. The decrease in the cadmium TF index is likely associated with increased immobilization of excess cadmium in the rhizosphere and potential modifications in root membrane permeability [34]. The higher cadmium content in *Zea mays* roots than in the aboveground parts, which was found in the present study, is confirmed by our previous research [38]. The higher cadmium content in *Zea mays* roots than in the aboveground parts, which was found in the present study, is confirmed by our previous research [80] and by reports from other researchers [64,84]. A similar relationship has also been demonstrated for *Sinapis alba* L. and *Avena sativa* L. [46].

Our research hypothesis, which was that the use of organic sorbents would lead to a significant reduction in cadmium uptake by *Zea mays*, thus mitigating the negative impact of cadmium on the plant’s growth and development as well as heavy metal accumulation, was only partially verified. Specifically, only the application of compost and HumiAgra significantly mitigated the toxic effects of cadmium. Regardless of the soil contamination level (15 and 30 mg Cd kg^−1^), compost increased aboveground yield by 31% and root biomass by 16% on average, while HumiAgra resulted in increases of 15% and 10%, respectively. In this respect, neither starch nor fermented bark met the expectations placed on them. It should also be emphasized that all organic sorbents, with the exception of starch, were able to mitigate the adverse effect of cadmium on leaf greenness. With regard to the biomass of aboveground parts and roots, compost and HumiAgra were the most effective, whereas starch and bark proved ineffective.

Therefore, strategies for mitigating the toxicity of Cd and other heavy metals in plants are an important topic [85]. Organic substances that can be used for this purpose include tobacco stems [86], rice husk [85,87], manure [88], biochar [38,44,87,88,89,90,91], humic acids [45,92], cellulose [46,93,94], wood biomass ash [95,96] and cereal straw [97]. The main remediation strategies identified by Mushtaq et al. [60] include physicochemical, phenolic, and phytohormone-assisted remediation. Abbas et al. [88] and Abd-El-Mageed et al. [90] attribute the positive effect of compost on plants grown in soil degraded by heavy metals to an improvement in soil fertility through changes in its physical, chemical, and microbiological properties. Biochar [38,98,99,100] and compost are widely recognized as sorbents [99,100]. Castellini et al. [99] and Mikajlo et al. [100] emphasize that biochar and compost affect soil properties in different yet often complementary ways. Compared to compost, biochar has a higher porosity and greater chemical stability. This improves soil physical properties by increasing water retention, air capacity, and aggregate stability. Compost is primarily a source of nutrients and improves soil structure, but its effects are short-lived. According to Mikajlo et al. [100], the best results are achieved when biochar and compost are applied simultaneously. In our studies, we opted to use compost due to its lower implementation cost. According to Ansar et al. [101], the cost of producing compost ranges from USD 20 to 50 per tonne, while that of biochar ranges from USD 300 to 1200 per tonne. Furthermore, we assumed that, as a good source of nutrients, compost could be an excellent aid for maize in meeting its nutritional needs, which may be disrupted in cadmium-contaminated soil.

Canal et al. [102] and Mushtaq et al. [60] observe that the addition of organic sorbents reduces cadmium toxicity, probably due to a “dilution” effect. In our own studies, both compost and HumiAgra were found to increase cadmium uptake from the soil. This is evident from an increase in the aboveground parts of the plant and a decrease in its roots, as well as from their impact on the yield of *Zea mays*. The positive effect of compost and HumiAgra on the growth and development of the tested plant is due to these two substances mitigating environmental stress. This is consistent with the research by Yang et al. [103], who demonstrated that the increase in cadmium phytoextraction by *Solanum nigrum* L. (*S. nigrum*) plants grown in contaminated soil supplemented with humic substances (fulvic acid or humic acid) was primarily due to an increase in biomass, rather than from an increase in Cd concentration in plant tissues.

Research by Evangelou et al. [104] showed that humic acids (HA) applied at a rate of 2 g kg^−1^ of soil increased Cd uptake by tobacco by as much as 65%. The authors attributed this finding to a decrease in pH and the formation of Cd–HA complexes, among other factors. The possibility that plants take up cadmium complexes with fragments of humic acids, formed through microbial degradation or spontaneous dissociation, was also considered. The study by Al Mamun et al. [105] showed that although Cd sorption in soil changes over time following compost application, the addition of fresh compost may increase the plant-available fraction of Cd, leading to greater accumulation in plant tissues during the early growth stage. Our results show an increase in the translocation of lead, chromium, copper, nickel, zinc, iron and manganese from the roots to the aboveground parts of plants.

Our own research has shown that cadmium contamination of the soil led to a significant, albeit slight, decrease in its pH, which may have contributed to increased cadmium mobility and its bioavailability to maize. Drabesch et al. [106] report that even a slight decrease in pH can significantly increase cadmium mobility and its bioavailability in soils with a pH below 6.0. At the same time, we demonstrated that compost and starch had a slightly alkalizing effect, whereas fermented bark had an acidifying effect. The acidifying effect of fermented bark results from the strongly acidic pH of the bark serving as the substrate for fermentation. The beneficial effect of compost on soil pH stems from the fact that it is rich in stable humic fractions and base cations, which increases the content of organic matter and nitrogen in the soil, as noted by Matisic et al. [107] and Nandillon et al. [108]. In our studies as well, compost increased the C_org_ and N_total_ content to the greatest extent. The lack of a significant effect of cadmium on C_org_ and N_total_ observed in our own studies confirms the concept that appropriately selected organic sorbents can stabilize soil function, which is under pressure not only from cadmium but also from other heavy metals.

The addition of organic sorbents to uncontaminated soil limited the transport of Cd and Fe (with the exception of compost), whereas under cadmium-induced stress conditions, all sorbents increased the translocation of Cd and Cu while reducing the movement of Fe and Ni (with the exception of bark at 30 mg Cd). Higher TF values in *Zea mays* grown in soil supplemented with organic sorbents confirm the high mobility and internal transfer of cadmium, as evidenced by a clear dose–response relationship [38].

Given that cadmium concentrations in European soils have been reported at levels up to 65.4 mg kg^−1^ by Ballabio et al. [24] and that Zulfiqar et al. [109] demonstrated that in agricultural soils these levels may reach 100 mg kg^−1^ dry matter, it was assumed that the application of 15 and 30 mg kg^−1^ dry soil represents a simulation of point-source contamination, thereby enabling the analysis of Cd effects on growth and metal accumulation in maize.

**Figure 11:**
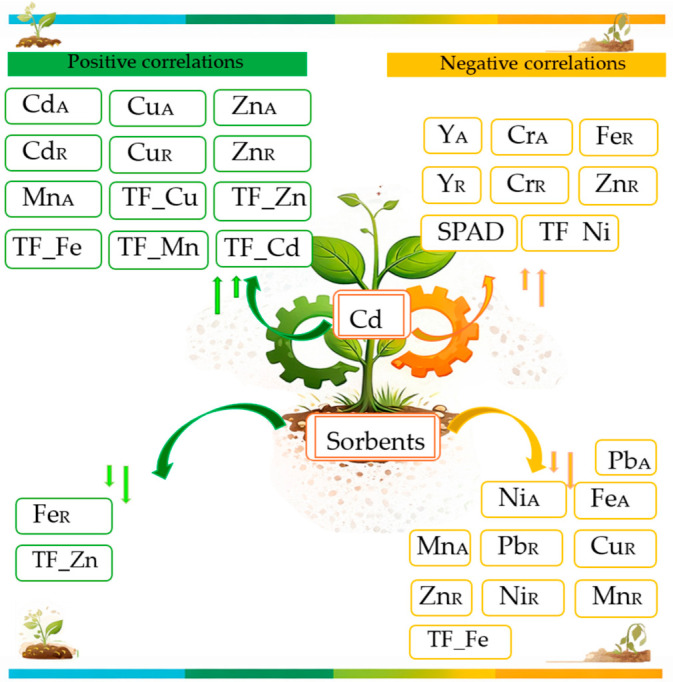
A graphical representation of the key findings from studies on the effects of cadmium and sorbents on *Zea mays*. Explanations: A—element content in aboveground parts, R—element content in roots, TF—element translocation index.

Our studies clearly demonstrate that cadmium contamination of soil at doses of 15 and 30 mg kg^−1^ adversely affects both the yield and ion homeostasis of *Zea mays*. Among the four sorbents tested, compost and HumiAgra mitigated this effect to the greatest extent, although neither completely eliminated it. In conclusion, and by comparing these considerations with the results of our study, it can be stated that the increased translocation of many heavy metals in the presence of excess Cd not only directly reduces the yield and ion homeostasis of *Zea mays* but also destabilizes the transport and distribution of many heavy metals, potentially exacerbating their phytotoxicity (Figure 11). Understanding these relationships is crucial for assessing soil health and developing phytoremediation strategies for areas contaminated with cadmium and other heavy metals.

## 4. Materials and Methods

### 4.1. Characteristics of the Soil, Plants, and Substances Supporting the Remediation Process

The soil used in the study was collected from the Masurian Lake District (Poland). It was a loamy sand consisting of sand (0.05–2 mm) at 63.61%, silt (0.002–0.05 mm) at 32.68%, and clay (<0.002 mm) at 3.71%. The soil contained 10.00 g C_org_ kg^−1^ of dry matter (d.m.), 0.83 g N_total_ kg^−1^ of d.m., a pH of 4.40 in 1 mol KCl dm^−3^, a pH of 5.52 in H_2_O dm^−3^, a hydrolytic acidity of − 26.10 mM(^+^) kg^−1^ d.m., exchangeable base cations of 63.60 mM(^+^) kg^−1^ d.m., a cation exchange capacity of 89.70 mM(^+^) kg^−1^ d.m., and an alkaline cation saturation of 70.90%. The soil contained the following amounts of heavy metals (mg kg^−1^ d.m.): Cd—0.14; Pb—17.35; Cr—46.02; Cu—13.56; Zn—55.45; Ni—10.09; Fe—12,654.55; Mn—233.63.

The study used maize (*Zea mays* L.) of the DS1897B variety. Maize cultivation has several advantages, including its widespread cultivation worldwide [110,111] and its ability to adapt to diverse habitat conditions. Furthermore, this species is characterized by increased tolerance to cadmium in the soil environment [112], and the biomass obtained can be effectively utilized in the energy sector [91,113].

To reduce cadmium toxicity, four substances were used to aid the remediation process: starch, compost, fermented coniferous bark, and the HumiAgra preparation.

Each of these sorbents had distinct physicochemical properties. The starch used in the study (manufacturer: Chempur, Piekary Śląskie, Poland) was a pure, white powder with the molecular formula (C_6_H_10_O_5_)ₙ and a molar mass of 162.1 g mol^−1^. It is characterized by moderate water solubility, amounting to 50 g dm^−3^ at 90 °C, with an optimal pH range of 6.0–7.5. Starch can be an important sorbent due to its widespread presence in the environment and its biopolymer structure, in which glucose molecules are linked by α-D-(1,4) and α-D-(1,6) bonds [114].

The compost was obtained from composted grass biomass. It contained: 252.76 g organic matter (OM); 146.61 g C_org_ kg^−1^ d.m., 20.18 g N_total_ kg^−1^ d.m., 3.41 mg P kg^−1^ d.m., 9.25 mg K kg^−1^ d.m., and 5.69 mg kg^−1^ d.m., and: 659.76—sum of exchangeable base cations (EBC); 82.04—hydrolytic acidity (HAC); 741.80—cation exchange capacity (CEC) (mmol (+) kg^−1^ d.m.); 88.94%—alkaline cation saturation (ACS). The pH of the compost was slightly acidic (pH_KCl_ = 6.1). The heavy metal content in the compost was as follows, in mg kg^−1^ d.m.: Cd—0.151; Pb—2.400; Cu—5.163; Zn—20.378; Ni—2.193; Cr—1.135; Fe—543.638; Mn—45.627.

The fermented bark (manufacturer: Athena Bio–Produkty Sp. z o.o., Golczewo, Poland) was derived from shredded coniferous wood. The material consisted of flakes measuring 20–50 mm, containing ≥30% dry matter and ≥50% organic matter. Its pH in KCl was 3.82. The adsorptive capacity of the bark is related to van der Waals interactions and the complex structure of its components, composed of p-hydroxyphenyl, guaiacyl, and syringyl units [52,115]. The heavy metal content in the fermented bark was as follows, in mg kg^−1^ d.m.: Cd—0.326; Pb—1.968; Cu—3.924; Zn—15.867; Ni—5.425; Cr—0.896; Fe—154.087; Mn—45.166.

HumiAgra (manufacturer: AgraPlant, Kielce, Poland) is an organic powder of dark brown color, containing 90% humic acids in a humin: fulvic ratio of 1:1. The product was characterized by an alkaline reaction (pH 8–10) and 8% K_2_O and 3% S. The detailed characteristics of the compost, fermented bark and HumiAgra used in this experiment can be found in our previous work [116,117,118]. The control was cadmium-contaminated soil without organic sorbents. Starch and HumiAgra did not contain any heavy metals.

### 4.2. Vegetation Experiment

The study was conducted under controlled conditions in a growth chamber located in northeastern Poland (53°45′36″ N, 20°27′15″ E). The experiment was set up in four replicates, using 16 cm high, 17 cm diameter polyethylene pots with a volume of 3.6 dm^3^. The bottom of each pot contained 12 holes, each 1.5 cm in diameter. Each pot was placed on an individual saucer. The air-dried soil, the characteristics of which are presented in Section 2.1, was sieved through a 5 mm mesh sieve prior to the experiment. It was then enriched with nutrients in the form of aqueous solutions of mineral fertilizers:

- Nitrogen (150 mg N kg^−1^ soil)—urea [CO(NH_2_)_2_] (Manufacturer: Stanlab, Lublin, Poland);
- Phosphorus (50 mg P kg^−1^ soil)—KH_2_PO_4_ (Manufacturer: Stanlab, Lublin, Poland);
- Potassium (150 mg K kg^−1^ soil)—KH_2_PO_4_ (Manufacturer: Stanlab, Lublin, Poland) + KCl (Manufacturer: Stanlab, Lublin, Poland);
- Magnesium (20 mg Mg kg^−1^ soil)—MgSO_4_·7H_2_O (Manufacturer: Stanlab, Lublin, Poland).

The next step was to divide the soil into three parts: 1—uncontaminated soil (control), 2—contaminated with 15 mg Cd kg^−1^ d.m., 3—contaminated with 30 mg Cd kg^−1^ d.m. Cadmium was introduced into the soil in the form of the salt CdSO_4_ 8H_2_O (Sigma Aldrich, St. Louis, Missouri, USA). Before filling the pots, the soil was thoroughly mixed separately for each pot with the fertilizers, and in the contaminated treatments, also with cadmium sulfate. One of four phytoremediation substances (starch, compost, fermented bark or HumiAgra) was added to each series, or the soil was left untreated (control).

The organic sorbents were added at a rate of 3 g of organic carbon per kg of soil. A total of 60 pots were prepared (5 sorbents × 3 cadmium levels × 4 replicates). The soil in the pots was then moistened to 60% of its maximum water-holding capacity, and this level was maintained throughout the duration of the experiment (55 days). Soil moisture was maintained by irrigation with demineralized water. On the day the experiment was set up, 8 seeds of *Zea mays* (variety DS1897B) were sown in each pot. After emergence, 4 plants were left in each pot. When the plants reached the Biologische Bundesanstalt, Bundessortenamt and Chemical Scale (BBCH) stage 51 (tassel initiation), the leaf greenness index (SPAD) was measured using a chlorophyll meter (Chlorophyll Meter, Spectrum Technologies, Konica Minolta, Tokyo, Japan). Subsequently, the aboveground parts and roots of the plants were harvested, and soil samples were collected for further laboratory analysis.

### 4.3. Laboratory Analysis Methods

The aboveground parts and roots of *Zea mays* were dried in a Binder D–78532 convection oven (Tuttlingen, Germany) at 60 °C until a constant weight was reached. Meanwhile, the soil samples were air-dried.

The following soil analyses were performed prior to the experiment:

- particle size distribution—using laser diffraction (Malvern Mastersizer 3000 Laser Diffraction (Malvern, Worcestershire, UK),
- C_org_ and N_total_ content—Vario MaxCube CN macroanalyzer (Elementar, Germany),
- soil pH—measured in 1 mol KCl and in water (pH meter HI 2221, Hanna Instruments, Washington, UK),
- hydrolytic acidity and total exchangeable base cations—according to the procedures described in previous works by the authors [91],

The content of cadmium, lead, chromium, copper, zinc, nickel, iron and manganese was determined using an inductively coupled plasma emission spectrometer (iCAP 7000 Series ICP–OES, Thermo Scientific, Newington, CT, USA).

These elements were also determined in the biomass of the aboveground parts and roots of *Zea mays* after harvest. To determine the content of cadmium, lead, chromium, copper, zinc, nickel, iron, and manganese in the soil, aboveground parts, and roots, soil and plant samples were subjected to digestion in a microwave digestion system (UltraWAVE, Milestone, Sorisole, Italy).

Mineralization was performed using 5 mL of 65% HNO_3_ per 0.5 g of sample, after which the material was diluted with demineralized water to a final volume of 100 mL. Analyses were performed using inductively coupled plasma emission spectrometry (iCAP 7000 Series ICP–OES, Thermo Scientific, Newington, CT, USA). All analyses were performed in quadruplicate. The study used a certified multi-element standard solution (Certipur^®^, Merck, Darmstadt, Germany), which contained 24 elements, including cadmium, lead, chromium, copper, zinc, nickel, iron and manganese.

### 4.4. Analysis of Results

The toxicity index, translocation index and heavy metal uptake were calculated based on the dry matter yield of *Zea mays* grown in cadmium-contaminated and uncontaminated soil, as well as the concentrations of cadmium, lead, chromium, copper, zinc, nickel, iron and manganese in the aboveground parts and roots. These calculations were performed using the formulas presented in Table 2. The heavy metal translocation index (TF) was calculated using the formula proposed by Smical et al. [119], while the toxicity index (TE) and heavy metals uptake by *Zea mays* (U) were calculated using formulas developed by the authors.

All the results presented in this study were statistically analyzed using Statistica 13.3 software [120]. The statistical analysis of the results aimed to assess the relationships between the studied variables (factor I—degree of cadmium contamination in the soil; factor II—type of organic sorbent). The distribution of the variables was assessed using the Shapiro–Wilk test. Homogeneous groups were identified using Tukey’s HSD test at a significance level of *p* = 0.01. In addition to statistical significance, the effect size measure η^2^ for ANOVA and Pearson’s correlation coefficients (*p* < 0.01) were presented for the studied variables. The results were presented in tables with three decimal places of accuracy (means ± SD), as well as in box plots and heat maps. The analysis was performed using Statistica 13.3, RStudio 2023.06.0 [121], with the R 4.2.2 add-on [122] and the gplots library [123], as well as the free online data visualization platform SRplot [124].

## 5. Conclusions

Cadmium added to clay loam at doses of 15 and 30 mg kg^−1^ d.m. of soil exhibits toxic effects on the root and aboveground growth of *Zea mays*. This disrupts normal plant growth and also alters the metabolism of metals such as lead, chromium, copper, zinc, nickel, iron, and manganese. Furthermore, it causes increased translocation of these elements from the roots to the aboveground parts. The application of organic sorbents to cadmium-uncontaminated soil leads to a reduction in cadmium translocation, while simultaneously increasing the movement of other metals. In cadmium-contaminated soil, however, these sorbents increase the translocation of copper and zinc, while reducing that of nickel and iron. Due to their ability to mitigate cadmium toxicity in plants, compost and HumiAgra are particularly recommended sorbents for remediation processes. Future studies should focus on the selection of appropriate sorbents for specific remediation methods (e.g., phytoremediation, rhizofiltration) and for the type of contaminant to be removed. These studies should be extended to include parallel analyses of not only the total cadmium content in the soil but also its speciation. Such research may contribute to a more detailed understanding of the mechanisms governing cadmium uptake by plants.

## Figures and Tables

**Table 1:** Uptake of heavy metals by *Zea mays*, µg per pot.

Treatment	Dose Cd, mg kg^–1^ Soil			Average
	0	15	30	
Cd				
Control (C)	1.253 ± 0.060 ^f A^	28.975 ± 2. 163 ^bc AB^	22.239 ± 2.368 ^e C^	17.489 ^II^
Starch (St)	0.813 ± 0.086 ^f B^	24.671 ± 3.798 ^cde B^	23.186 ± 2.002 ^de C^	16.223 ^II^
Compost (Cm)	0.713 ± 0.140 ^f B^	34.492 ± 3.418 ^ab A^	38.200 ± 0.554 ^a A^	24.468 ^I^
Bark (B)	1.111 ± 0.024 ^f A^	28.085 ± 1.018 ^cd B^	26.418 ± 3.671 ^cde BC^	18.538 ^II^
HumiAgra (HA)	0.687 ± 0.037 ^f B^	23.719 ± 3.356 ^cde B^	29.087 ± 3.109 ^bc B^	17.831 ^II^
Average	0.915 ^Y^	27.988 ^Z^	27.826 ^Z^	
Source of variation (F statistic and probability level): Cd dose: F—956.37 (*p* < 0.01); Treatment: F—24.52 (*p* < 0.01); Cd dose x Treatment: F—11.07 (*p* < 0.01)				
Pb				
Control (C)	57.130 ± 1.889 ^a A^	32.304 ± 1.339 ^d B^	7.374 ± 0.811 ^h C^	32.269 ^II^
Starch (St)	45.278 ± 2.661 ^b B^	20.658 ± 1.586 ^f D^	7.107 ± 0.232 ^h C^	24.348 ^III^
Compost (Cm)	53.382 ± 2.483 ^a A^	37.228 ± 2.335 ^c A^	22.752 ± 0529 ^ef A^	37.787 ^I^
Bark (B)	53.287 ± 2.778 ^a A^	19.883 ± 1.330 ^f D^	5.037 ± 1.193 ^h D^	26.069 ^III^
HumiAgra (HA)	20.882 ± 2.476 ^f C^	25.536 ± 1.457 ^e C^	14.408 ± 0.960 ^g B^	20.275 ^IV^
Average	45.992 ^Z^	27.122 ^Y^	11.335 ^X^	
Source of variation (F statistic and probability level): Cd dose: F—1894.34 (*p* < 0.01); Treatment: F—179.83 (*p* < 0.01); Cd dose x Treatment: F—115.52 (*p* < 0.01)				
Cr				
Control (C)	359.919 ± 18.014 ^b B^	98.464 ± 8.352 ^efg AB^	29.942 ± 2.374 ^ij C^	162.775 ^II^
Starch (St)	116.998 ± 8.855 ^e E^	57.350 ± 6.118 ^h D^	20.232 ± 1.991 ^j D^	64.860 ^V^
Compost (Cm)	220.249 ± 10.685 ^c C^	108.713 ± 14.043 ^ef A^	51.279 ± 2.929 ^hi A^	126.747 ^III^
Bark (B)	412.048 ± 24.300 ^a A^	89.206 ± 2.802 ^fg BC^	29.201 ± 2.986 ^j C^	176.818 ^I^
HumiAgra (HA)	180.798 ± 9.314 ^d D^	80.160 ± 6.159 ^g C^	37.922 ± 1.734 ^hij B^	99.627 ^IV^
Average	258.002 ^Z^	86.779 ^Y^	33.715 ^X^	
Source of variation (F statistic and probability level): Cd dose: F—3869.26 (*p* < 0.01); Treatment: F—354.52 (*p* < 0.01); Cd dose x Treatment: F—269.88 (*p* < 0.01)				
Cu				
Control (C)	178.423 ± 6.062 ^a A^	80.685 ± 4.107 ^e B^	41.038 ± 2.628 ^h C^	100.049 ^II^
Starch (St)	131.147 ± 7.431 ^c C^	57.794 ± 6.818 ^g D^	30.561 ± 3.115 ^h D^	73.167 ^IV^
Compost (Cm)	144.356 ± 8.730 ^b B^	124.816 ± 5.554 ^c A^	105.325 ± 6.140 ^d A^	124.832 ^I^
Bark (B)	179.343 ± 3.475 ^a A^	76.060 ± 3.618 ^ef BC^	40.824 ± 3.841 ^h C^	98.742 ^II^
HumiAgra (HA)	105.394 ± 1.231 ^d D^	69.237 ± 2.870 ^efg C^	63.681 ± 2.206 ^fg B^	79.437 ^III^
Average	147.733 ^Z^	81.718 ^Y^	56.286 ^Z^	
Source of variation (F statistic and probability level): Cd dose: F—1796.39 (*p* < 0.01); Treatment: F—200.67 (*p* < 0.01); Cd dose x Treatment: F—106.91 (*p* < 0.01)				
Zn				
Control (C)	1380.955 ± 47.795 ^c C^	744.505 ± 12.990 ^g B^	332.908 ± 28.297 ^k C^	819.456 ^II^
Starch (St)	936.598 ± 58.891 ^f D^	468.714 ± 52.567 ^j D^	228.513 ± 26.219 ^k D^	544.608 ^IV^
Compost (Cm)	1955.927 ± 27.858 ^a A^	1262.289 ± 64.762 ^d A^	1115.209 ± 53.493 ^e A^	1444.475 ^I^
Bark (B)	1521.578 ± 36.248 ^b B^	732.215 ± 29.677 ^gh B^	319.811 ± 37.587 ^k C^	857.868 ^II^
HumiAgra (HA)	954.903 ± 54.554 ^f D^	585.432 ± 76.500 ^i C^	622.326 ± 20.684 ^hi B^	720.887 ^III^
Average	1349.992 ^Z^	758.631 ^Y^	523.753 ^X^	
Source of variation (F statistic and probability level): Cd dose: F—1761.62 (*p* < 0.01); Treatment: F—671.52 (*p* < 0.01); Cd dose x Treatment: F—57.38 (*p* < 0.01)				
Ni				
Control (C)	236.217 ± 10.894 ^a A^	110.901 ± 0.752 ^e A^	38.299 ± 2.737 ^hi C^	128.472 ^I^
Starch (St)	103.554 ± 4.481 ^e E^	61.400 ± 3.828 ^g B^	24.378 ± 1.619 ^j D^	63.111 ^V^
Compost (Cm)	152.140 ± 2.185 ^c B^	107.180 ± 6.725 ^e A^	74.116 ± 2.023 ^f A^	111.145 ^II^
Bark (B)	199.108 ± 9.353 ^b B^	67.395 ± 1.024 ^fg B^	34.122 ± 2.269 ^ij C^	100.208 ^III^
HumiAgra (HA)	134.491 ± 4.788 ^d D^	69.803 ± 4.087 ^fg B^	47.778 ± 2.294 ^h B^	84.024 ^IV^
Average	165.102 ^Z^	83.336 ^Y^	43.738 ^X^	
Source of variation (F statistic and probability level): Cd dose: F—3220.68 (*p* < 0.01); Treatment: F—316.88 (*p* < 0.01); Cd dose x Treatment: F—152.58 (*p* < 0.01)				
Fe				
Control (C)	33,651.593 ± 1555.263 ^b B^	10,170.735 ± 257.548 ^gh CD^	4783.155 ± 361.386 ^j C^	16,201.827 ^II^
Starch (St)	14,623.211 ± 471.824 ^f D^	8912.132 ± 481.665 ^h D^	3032.663 ± 230.238 ^j E^	8856.002 ^III^
Compost (Cm)	27,382.263 ± 247.786 ^d C^	17,748.049 ± 811.667 ^e A^	9686.012 ± 122.297 ^gh A^	18,272.108 ^I^
Bark (B)	36,982.195 ± 1641.235 ^a A^	11,352.547 ± 134.515 ^g C^	4108.865 ± 125.275 ^j D^	17,481.202 ^I^
HumiAgra (HA)	31,195.475 ± 1310.275 ^c B^	15,086.661 ± 1099.373 ^f B^	6157.917 ± 432.782 ^I B^	17,480.017 ^I^
Average	28,766.947 ^Z^	12,654.025 ^Y^	5553.722 ^X^	
Source of variation (F statistic and probability level): Cd dose: F—4378.79 (*p* < 0.01); Treatment: F—278.42 (*p* < 0.01); Cd dose x Treatment: F—154.58 (*p* < 0.01)				
Mn				
Control (C)	4988.153 ± 148.305 ^a B^	3216.047 ± 17.864 ^b B^	1240.502 ± 115.143 ^d BC^	3148.234 ^II^
Starch (St)	3196.570 ± 187.200 ^b C^	3131.340 ± 330.038 ^b B^	1044.700 ± 107.797 ^d D^	2457.536 ^III^
Compost (Cm)	5282.130 ± 81.192 ^a AB^	5068.639 ± 314.562 ^a A^	3246.746 ± 131.861 ^b A^	4532.505 ^I^
Bark (B)	5390.533 ± 114.082 ^a A^	3314.737 ± 176.298 ^b B^	1271.757 ± 104.448 ^d BC^	3325.676 ^II^
HumiAgra (HA)	3208.080 ± 221.860 ^b C^	2486.622 ± 99.121 ^c C^	1425.686 ± 99.121 ^d B^	2373.463 ^III^
Average	4413.093 ^Z^	3443.477 ^Y^	1645.878 ^X^	
Source of variation (F statistic and probability level): Cd dose: F—1334.50 (*p* < 0.01); Treatment: F—306.82 (*p* < 0.01); Cd dose x Treatment: F—49.07 (*p* < 0.01)				

Lowercase letters indicate the significance of differences for both variables, i.e., Cd dose and sorbent addition; uppercase letters indicate the significance of differences for sorbent type separately for each Cd dose; Roman numerals indicate the significance of differences for mean sorbent results, regardless of the cadmium dose; the letters X, Y, and Z indicate the significance of differences for the mean Cd dose results, regardless of sorbent addition.

**Table 2:** Formulas for calculating cadmium toxicity, heavy metal translocation, and heavy metal uptake from the soil.

Parameter	Formula	Explanations
Cadmium toxicity index (TE)	$\mathrm{TE}\;(\%)=\frac{Yc-Y_{Cd}}{Y_{Cd}}$ × 100%	Y_C—_SPAD/yield of *Zea mays* in uncontaminated (control) soil Y_Cd—_SPAD/*Zea mays* yield in cadmium-contaminated soil
Heavy metal translocation index (TF)	$TF=\frac{{Me}_{A}}{{Me}_{R}}$	Me_A_—heavy metal content in the aboveground parts of *Zea mays* Me_R_—heavy metal content in the roots of *Zea mays*
Heavy metal uptake by *Zea mays* (U)	U_A_ = Y_A_ × Me_A_ + Y_R_ × Me_R_	Y_A—_yield of aboveground parts of *Zea mays* Me_A_—heavy metal content in aboveground parts of *Zea mays* Me_R_—heavy metal content in roots of *Zea mays*

## Data Availability

The original contributions presented in this study are included in the article. Further inquiries can be directed to the corresponding author.

## Abbreviations

The following abbreviations are used in this manuscript:

0	Soil uncontaminated with cadmium
C	Soil not fertilized with organic sorbents
Cd	Soil contaminated with cadmium
St	Soil fertilized with starch
Cm	Soil fertilized with compost
B	Soil fertilized with fermented starch
HA	Soil fertilized with HumiAgra
Zm	*Zea mays*
A	Aboveground of *Zea mays*
R	Roots of *Zea mays*
YA	Aboveground yield of *Zea mays*
YR	Root yield of *Zea mays*
YC	Yield of *Zea mays* in uncontaminated soil (control)
YCd	Yield of *Zea mays* in cadmium-contaminated soil
SPAD	Leaf greenness index
MeA	Heavy metal content in the aboveground parts of *Zea mays*
MeR	Heavy metal content in *Zea mays* roots
TE	Cadmium toxicity index
TF	Heavy metal translocation index
U	Heavy metal uptake by *Zea mays*

## Appendix A

**Table A1:** Heavy metal content in the aboveground parts of *Zea mays*, mg kg^1^ d.m.

Treatment	Dose Cd, mg kg^–1^ Soil			Average
	0	15	30	
Cd				
Control (C)	0.015 ± 0.001 ^h A^	0.300 ± 0.044 ^g C^	0.923 ± 0.002 ^d D^	0.413 ^V^
Starch (St)	0.010 ± 0.001 ^h B^	0.833 ± 0.001 ^e A^	2.185 ± 0.008 ^a A^	1.009 ^I^
Compost (Cm)	0.005 ± 0.000 ^h D^	0.445 ± 0.009 ^f B^	0.894 ± 0.002 ^d E^	0.448 ^IV^
Bark (B)	0.008 ± 0.001 ^h C^	0.830 ± 0.002 ^e A^	1.892 ± 0.001 ^b B^	0.910 ^II^
HumiAgra (HA)	0.003 ± 0.001 ^h E^	0.474 ± 0.002 ^f B^	1.137 ± 0.001 ^c C^	0.538 ^III^
Average	0.008 ^X^	0.576 ^Y^	1.406 ^Z^	
Source of variation (F statistic and probability level): Cd dose: F—70,524.65 (*p* < 0.01); Treatment: F—6533.69 (*p* < 0.01); Cd dose x Treatment: F—2598.74 (*p* < 0.01)				
Pb				
Control (C)	0.750 ± 0.058 ^cd B^	1.400 ± 0.041 ^a A^	0.550 ± 0.100 ^de B^	0.900 ^I–II^
Starch (St)	1.000 ± 0.041 ^bc A^	1.150 ± 0.100 ^ab A^	0.800 ± 0.041 ^cd A^	0.983 ^I^
Compost (Cm)	0.800 ± 0.041 ^cd B^	0.850 ± 0.058 ^cd B^	0.800 ± 0.041 ^cd A^	0.817 ^II–III^
Bark (B)	0.750 ± 0.058 ^cd B^	0.800 ± 0.082 ^cd B^	0.350 ± 0.058 ^ef C^	0.633 ^IV^
HumiAgra (HA)	0.250 ± 0.058 ^f C^	1.150 ± 0.100 ^ab A^	0.850 ± 0.058 ^cd A^	0.750 ^III^
Average	0.710 ^Y^	1.070 ^X^	0.670 ^X^	
Source of variation (F statistic and probability level): Cd dose: F—281.81 (*p* < 0.01); Treatment: F—63.39 (*p* < 0.01); Cd dose x Treatment: F—74.71 (*p* < 0.01)				
Cr				
Control (C)	4.200 ± 0.566 ^b B^	3.400 ± 0.163 ^c A^	2.800 ± 0.163 ^cdef A^	3.467 ^II^
Starch (St)	2.200 ± 0.041 ^ef D^	2.750 ± 0.191 ^def B^	2.050 ± 0.100 ^gh C^	2.333 ^III^
Compost (Cm)	2.850 ± 0.191 ^de C^	1.950 ± 0.191 ^h C^	1.200 ± 0.082 ^i E^	2.000 ^III–IV^
Bark (B)	6.550 ± 0.252 ^a A^	3.300 ± 0.183 ^cd A^	2.575 ± 0.206 ^efg B^	4.142 ^I^
HumiAgra (HA)	1.850 ± 0.100 ^g D^	2.750 ± 0.191 ^def B^	1.850 ± 0.100 ^h D^	2.150 ^V^
Average	3.530 ^Z^	2.830	2.095 ^X^	
Source of variation (F statistic and probability level): Cd dose: F—230.27 (*p* < 0.01); Treatment: F—236.33 (*p* < 0.01); Cd dose x Treatment: F—78.01 (*p* < 0.01)				
Cu				
Control (C)	2.950 ± 0.100 ^de A^	3.400 ± 0.283 ^cd A^	4.050 ± 0.100 ^ab AB^	3.467 ^I^
Starch (St)	2.400 ± 0.041 ^fg B^	2.900 ± 0.200 ^e B^	3.750 ± 0.100 ^bc B^	3.017 ^II^
Compost (Cm)	2.200 ± 0.283 ^g B^	3.000 ± 0.163 ^de B^	3.800 ± 0.163 ^abc B^	3.000 ^II^
Bark (B)	2.800 ± 0.283 ^ef A^	3.250 ± 0.379 ^de A^	4.250 ± 0.100 ^a A^	3.433 ^I^
HumiAgra (HA)	1.600 ± 0.163 ^h C^	2.950 ± 0.100 ^de B^	3.950 ± 0.100 ^ab AB^	2.833 ^II^
Average	2.390 ^Y^	3.100 ^X^	3.960 ^Z^	
Source of variation (F statistic and probability level): Cd dose: F—357.74 (*p* < 0.01); Treatment: F—27.88 (*p* < 0.01); Cd dose x Treatment: F—7.61 (*p* < 0.01)				
Zn				
Control (C)	17.400 ± 0.082 ^l D^	34.850 ± 0.100 ^e A^	32.600 ± 0.100 ^g E^	28.283 ^IV^
Starch (St)	17.950 ± 0.100 ^k C^	29.500 ± 0.115 ^h C^	35.900 ± 0.200 ^d D^	27.783 ^V^
Compost (Cm)	29.000 ± 0.082 ^I A^	34.100 ± 0.115 ^f B^	41.750 ± 0.100 ^b B^	34.950 ^I^
Bark (B)	21.050 ± 0.100 ^j B^	35.050 ± 0.100 ^e A^	37.200 ± 0.082 ^c C^	31.100 ^II^
HumiAgra (HA)	15.200 ± 0.163 ^m E^	29.750 ± 0.191 ^h C^	42.800 ± 0.163 ^a A^	29.250 ^III^
Average	20.120 ^X^	32.650 ^Y^	38.050 ^Z^	
Source of variation (F statistic and probability level): Cd dose: F—11,1980.69 (*p* < 0.01); Treatment: F—6702.75 (*p* < 0.01); Cd dose x Treatment: F—3874.04 (*p* < 0.01)				
Ni				
Control (C)	2.700 ± 0.115 ^b A^	4.000 ± 0.041 ^a A^	2.350 ± 0.100 ^bcd A^	3.017 ^I^
Starch (St)	1.650 ± 0.100 ^g BC^	2.100 ± 0.115 ^cde B^	2.000 ± 0.082 ^defg AB^	1.917 ^III^
Compost (Cm)	2.000 ± 0.041 ^defg B^	1.850 ± 0.191 ^efg C^	1.800 ± 0.082 ^efg B^	1.883 ^III^
Bark (B)	2.500 ± 0.115 ^bc A^	1.750 ± 0.058 ^efg C^	2.150 ± 0.100 ^cde AB^	2.133 ^II^
HumiAgra (HA)	1.800 ± 0.041 ^efg BC^	1.750 ± 0.100 ^efg C^	1.850 ± 0.100 ^efg B^	1.800 ^III^
Average	2.130 ^Y^	2.290 ^Z^	2.030 ^X^	
Source of variation (F statistic and probability level): Cd dose: F—35.18 (*p* < 0.01); Treatment: F—306.65 (*p* < 0.01); Cd dose x Treatment: F—89.98 (*p* < 0.01)				
Fe				
Control (C)	132.850 ± 0.100 ^def A^	148.550 ± 3.206 ^c A^	220.550 ± 2.734 ^a A^	167.317 ^I^
Starch (St)	66.950 ± 3.017 ^I C^	111.250 ± 4.620 ^g D^	131.950 ± 2.357 ^def D^	103.383 ^V^
Compost (Cm)	129.800 ± 0.283 ^ef A^	136.600 ± 3.960 ^de B^	163.650 ± 5.468 ^b B^	143.350 ^II^
Bark (B)	86.200 ± 1.697 ^h B^	124.450 ± 4.190 ^f C^	166.400 ± 1.980 ^b B^	125.683 ^III^
HumiAgra (HA)	61.650 ± 2.169 ^I D^	134.900 ± 4.337 ^de BC^	141.200 ± 3.394 ^cd C^	112.583 ^IV^
Average	95.490 ^X^	131.150 ^Y^	164.750 ^Z^	
Source of variation (F statistic and probability level): Cd dose: F—2265.90 (*p* < 0.01); Treatment: F—736.70 (*p* < 0.01); Cd dose x Treatment: F—111.16 (*p* < 0.01)				
Mn				
Control (C)	70.550 ± 0.661 ^jk C^	145.000 ± 3.266 ^c E^	136.250 ± 1.509 ^e C^	117.267 ^III^
Starch (St)	67.100 ± 0.476 ^k D^	213.750 ± 0.943 ^a A^	145.400 ± 1.470 ^c A^	142.083 ^I^
Compost (Cm)	79.150 ± 0.473 ^i A^	126.150 ± 0.755 ^f C^	110.800 ± 0.653 ^h D^	105.367 ^IV^
Bark (B)	72.750 ± 0.661 ^j B^	153.350 ± 2.169 ^b B^	140.150 ± 1.603 ^d B^	122.083 ^II^
HumiAgra (HA)	47.450 ± 1.320 ^l E^	120.300 ± 0.115 ^g D^	80.750 ± 1.038 ^I E^	82.833 ^V^
Average	67.400 ^X^	151.710 ^Z^	122.670 ^Y^	
Source of variation (F statistic and probability level): Cd dose: F—19,363.60 (*p* < 0.01); Treatment: F—3025.16 (*p* < 0.01); Cd dose x Treatment: F—866.75 (*p* < 0.01)				

Lowercase letters indicate the significance of differences for both variables, i.e., Cd dose and sorbent addition; uppercase letters indicate the significance of differences for sorbent type separately for each Cd dose; Roman numerals indicate the significance of differences for mean sorbent results, regardless of the cadmium dose; the letters X, Y, and Z indicate the significance of differences for the mean Cd dose results, regardless of sorbent addition.

**Table A2:** Heavy metal contents in the roots of *Zea mays*, mg kg^1^ d.m.

Treatment	Dose Cd, mg kg^–1^ Soil			Average
	0	15	30	
Cd				
Control (C)	0.050 ± 0.004 ^j AB^	5.512 ± 0.096 ^c A^	6.151 ± 0.011 ^b B^	3.904 ^I^
Starch (St)	0.052 ± 0.001 ^j A^	3.569 ± 0.009 ^g B^	6.426 ± 0.007 ^a A^	3.349 ^II^
Compost (Cm)	0.039 ± 0.012 ^j B^	2.884 ± 0.115 ^I C^	3.687 ± 0.002 ^f E^	2.203 ^V^
Bark (B)	0.060 ± 0.001 ^j A^	3.002 ± 0.005 ^h C^	5.085 ± 0.010 ^d C^	2.715 ^III^
HumiAgra (HA)	0.056 ± 0.001 ^j A^	3.004 ± 0.010 ^h C^	4.342 ± 0.004 ^e D^	2.467 ^IV^
Average	0.051 ^X^	3.594 ^Y^	5.138 ^Z^	
Source of variation (F statistic and probability level): Cd dose: F—87,853.40 (*p* < 0.01); Treatment: F—3707.43 (*p* < 0.01); Cd dose x Treatment: F—1476.16 (*p* < 0.01)				
Pb				
Control (C)	2.000 ± 0.082 ^a A^	1.700 ± 0.,82 ^ab A^	1.400 ± 0.016 ^bc A^	1.700 ^I^
Starch (St)	1.100 ± 0.082 ^cd AB^	1.600 ± 0.163 ^b A^	1.600 ± 0.082 ^b A^	1.433 ^II^
Compost (Cm)	0.950 ± 0.058 ^de B^	1.600 ± 0.041 ^b A^	0.750 ± 0.100 ^e BC^	1.100 ^III^
Bark (B)	1.400 ± 0.082 ^bc B^	1.250 ± 0.100 ^cd B^	1.000 ± 0.065 ^de B^	1.217 ^III^
HumiAgra (HA)	1.000 ± 0.041 ^de B^	1.450 ± 0.100 ^bc AB^	1.050 ± 0.058 ^de B^	1.167 ^III^
Average	1.290 ^Y^	1.520 ^Z^	1.160 ^X^	
Source of variation (F statistic and probability level): Cd dose: F—117.94 (*p* < 0.01); Treatment: F—127.64 (*p* < 0.01); Cd dose x Treatment: F—54.36 (*p* < 0.01)				
Cr				
Control (C)	14.800 ± 0.712 ^a A^	8.800 ± 0.653 ^bc A^	4.200 ± 0.245 ^h B^	9.267 ^I^
Starch (St)	4.400 ± 0.141 ^gh E^	5.800 ± 0.327 ^efgh C^	5.200 ± 0.163 ^fgh A^	5.133 ^IV^
Compost (Cm)	6.000 ± 0.327 ^efg D^	6.850 ± 0.173 ^de B^	4.950 ± 0.526 ^gh A^	5.933 ^III^
Bark (B)	7.800 ± 0.432 ^bc C^	6.800 ± 0.408 ^def B^	4.250 ± 0.574 ^h B^	6.283 ^III^
HumiAgra (HA)	10.050 ± 0.342 ^b B^	6.950 ± 0.100 ^de B^	4.250 ± 0.342 ^h B^	7.083 ^II^
Average	8.610 ^Z^	7.040 ^Y^	4.570 ^X^	
Source of variation (F statistic and probability level): Cd dose: F—547.38 (*p* < 0.01); Treatment: F—196.61 (*p* < 0.01); Cd dose x Treatment: F—136.50 (*p* < 0.01)				
Cu				
Control (C)	3.700 ± 0.200 ^fg B^	4.650 ± 0.342 ^d B^	5.200 ± 0.163 ^bc B^	4.517 ^II^
Starch (St)	5.200 ± 0.163 ^bc A^	5.450 ± 0.100 ^b A^	6.000 ± 0.163 ^a A^	5.550 ^I^
Compost (Cm)	2.400 ± 0.163 ^i C^	4.750 ± 0.173 ^cd B^	2.950 ± 0.191 ^h D^	3.367 ^IV^
Bark (B)	3.600 ± 0.163 ^fg B^	4.000 ± 0.327 ^efg C^	4.100 ± 0.258 ^ef C^	3.900 ^III^
HumiAgra (HA)	3.550 ± 0.100 ^g B^	4.400 ± 0.283 ^de BC^	3.900 ± 0.115 ^C^	3.950 ^III^
Average	3.690 ^X^	4.650 ^Z^	4.430 ^Y^	
Source of variation (F statistic and probability level): Cd dose: F—117.95 (*p* < 0.01); Treatment: F—192.61 (*p* < 0.01); Cd dose x Treatment: F—28.96 (*p* < 0.01)				
Zn				
Control (C)	51.400 ± 0.245 ^a A^	28.400 ± 0.163 ^h B^	42.850 ± 0.100 ^b A^	40.883 ^I^
Starch (St)	33.850 ± 0.191 ^e D^	25.850 ± 0.100 ^i C^	22.550 ± 0.100 ^l C^	27.417 ^IV^
Compost (Cm)	36.250 ± 0.100 ^d C^	32.650 ± 0.100 ^f A^	23.050 ± 0.100 ^k B^	30.650 ^II^
Bark (B)	41.450 ± 0.100 ^c B^	23.000 ± 0.245 ^k E^	21.050 ± 0.100 ^n E^	28.500 ^III^
HumiAgra (HA)	29.050 ± 0.100 ^g E^	23.800 ± 0.082 ^j D^	22.000 ± 0.245 ^m D^	24.950 ^V^
Average	38.400 ^A^	26.740 ^B^	26.300 ^C^	
Source of variation (F statistic and probability level): Cd dose: F—41,552.82 (*p* < 0.01); Treatment: F—20,136.65 (*p* < 0.01); Cd dose x Treatment: F—5300.87 (*p* < 0.01)				
Ni				
Control (C)	9.950 ± 0.191 ^a A^	9.200 ± 0.490 ^ab A^	8.600 ± 0.490 ^bcd A^	9.250 ^I^
Starch (St)	5.100 ± 0.115 ^j D^	8.800 ± 0.653 ^bc AB^	7.600 ± 0.490 ^efg B^	7.167 ^II–III^
Compost (Cm)	4.000 ± 0.163 ^k E^	7.050 ± 0.100 ^fgh D^	6.800 ± 0.082 ^gh B^	5.950 ^IV^
Bark (B)	6.450 ± 0.252 ^hi B^	8.200 ± 0.327 ^cde BC^	7.400 ± 0.653 ^efg B^	7.350 ^II^
HumiAgra (HA)	5.600 ± 0.082 ^ij C^	7.850 ± 0.129 ^def CD^	7.200 ± 0.163 ^fgh B^	6.883 ^III^
Average	6.220 ^X^	8.220 ^Z^	7.520 ^Y^	
Source of variation (F statistic and probability level): Cd dose: F—166.13 (*p* < 0.01); Treatment: F—140.73 (*p* < 0.01); Cd dose x Treatment: F—31.02 (*p* < 0.01)				
Fe				
Control (C)	2472.550 ± 43.370 ^c C^	1754.400 ± 48.990 ^e C^	1265.200 ± 48.990 ^gh A^	1830.717 ^III^
Starch (St)	1393.750 ± 32.631 ^f E^	1871.200 ± 48.990 ^e C^	1277.200 ± 48.990 ^fgh A^	1514.050 ^V^
Compost (Cm)	1782.200 ± 35.803 ^e D^	1862.000 ± 20.537 ^e C^	1275.600 ± 20.493 ^fgh A^	1639.933 ^IV^
Bark (B)	2722.350 ± 61.105 ^b B^	2083.200 ± 97.980 ^d B^	1153.200 ± 65.320 ^h B^	1986.250 ^II^
HumiAgra (HA)	2874.050 ± 43.240 ^a A^	2375.300 ± 65.320 ^c A^	1301.200 ± 22.221 ^fg A^	2183.517 ^I^
Average	2248.980 ^Z^	1989.220 ^Y^	1254.480 ^X^	
Source of variation (F statistic and probability level): Cd dose: F—2198.82 (*p* < 0.01); Treatment: F—353.72 (*p* < 0.01); Cd dose x Treatment: F—209.66 (*p* < 0.01)				
Mn				
Control (C)	153.400 ± 2.304 ^bc A^	145.800 ± 2286 ^c B^	120.950 ± 1.935 ^de AB^	140.050 ^I^
Starch (St)	91.850 ± 1.518 ^g C^	121.550 ± 1.893 ^de C^	156.250 ± 1.320 ^b A^	123.217 ^III^
Compost (Cm)	94.050 ± 2.369 ^g C^	175.200 ± 2.718 ^a A^	125.200 ± 2.514 ^d B^	131.483 ^II^
Bark (B)	154.200 ± 2.998 ^b A^	126.050 ± 8.580 ^d C^	105.750 ± 1.389 ^f D^	128.667 ^II^
HumiAgra (HA)	113.600 ± 2.833 ^ef B^	118.000 ± 2.628 ^de C^	116.800 ± 3.254 ^e C^	116.133 ^IV^
Average	121.420 ^X^	137.320 ^Z^	124.990 ^Y^	
Source of variation (F statistic and probability level): Cd dose: F—138.03 (*p* < 0.01); Treatment: F—95.62 (*p* < 0.01); Cd dose x Treatment: F—322.48 (*p* < 0.01)				

Lowercase letters indicate the significance of differences for both variables, i.e., Cd dose and sorbent addition; uppercase letters indicate the significance of differences for sorbent type separately for each Cd dose; Roman numerals indicate the significance of differences for mean sorbent results, regardless of the cadmium dose; the letters X, Y, and Z indicate the significance of differences for the mean Cd dose results, regardless of sorbent addition.

**Table A3:** The effects of cadmium and sorbents on soil pH and carbon and nitrogen content.

Treatment	Dose Cd, mg kg^–1^ Soil			Average
	0	15	30	
pH_KCl_				
Control (C)	4.469 ± 0.025 ^e D^	4.469 ± 0.025 ^e B^	4.453 ± 0.012 ^e C^	4.463 ^III^
Starch (St)	4.521 ± 0.006 ^d C^	4.468 ± 0.001 ^e B^	4.527 ± 0.010 ^cd B^	4.505 ^II^
Compost (Cm)	4.724 ± 0.012 ^a A^	4.709 ± 0.010 ^a A^	4.643 ± 0.013 ^b A^	4.692 ^I^
Bark (B)	4.321 ± 0.009 ^g E^	4.272 ± 0.006 ^h D^	4.270 ± 0.021 ^h D^	4.288 ^IV^
HumiAgra (HA)	4.570 ± 0.002 ^c B^	4.366 ± 0.028 ^f C^	4.444 ± 0.006 ^e C^	4.460 ^III^
Average	4.521 ^Z^	4.457 ^Y^	4.467 ^Y^	
Source of variation (F statistic and probability level): Cd dose: F—80.59 (*p* < 0.01); Treatment: F—854.51 (*p* < 0.01); Cd dose x Treatment: F—67.97 (*p* < 0.01)				
C_org_, g kg^−1^ soil				
Control (C)	10.568 ± 0.173 ^a B^	10.561 ± 0.061 ^a B^	10.522 ± 0.098 ^a B^	10.550 ^III^
Starch (St)	13.069 ± 0.932 ^b A^	13.262 ± 0.262 ^b A^	13.143 ± 0.358 ^b A^	13.158 ^I–II^
Compost (Cm)	13.570 ± 0.200 ^b A^	13.459 ± 0.199 ^b A^	13.321 ± 0.201 ^b A^	13.450 ^I^
Bark (B)	12.974 ± 0.144 ^b A^	13.060 ± 0.060 ^b A^	12.819 ± 0.182 ^b A^	12.951 ^II^
HumiAgra (HA)	13.092 ± 0.098 ^b A^	13.082 ± 0.102 ^b A^	13.110 ± 0.110 ^b A^	13.095 ^I–II^
Average	12.654 ^Z^	12.685 ^Z^	12.583 ^Z^	
Source of variation (F statistic and probability level): Cd dose: F—0.47 (*p* = 0.63); Treatment: F—143.45 (*p* < 0.01); Cd dose x Treatment: F—0.23 (*p* < 0.23)				
N_total_, g kg^−1^ soil				
Control (C)	0.852 ± 0.012 ^de C^	0.852 ± 0.030 ^de C^	0.831 ± 0.031 ^e D^	0.845 ^V^
Starch (St)	0.922 ± 0.022 ^de C^	0.942 ± 0.022 ^cd B^	0.933 ± 0.033 ^d C^	0.932 ^IV^
Compost (Cm)	1.130 ± 0.070 ^a A^	1.109 ± 0.009 ^ab A^	1.121 ± 0.021 ^a A^	1.120 ^I^
Bark (B)	1.026 ± 0.015 ^bc B^	1.041 ± 0.041 ^ab A^	1.029 ± 0.029 ^bc B^	1.032 ^III^
HumiAgra (HA)	1.095 ± 0.007 ^ab AB^	1.066 ± 0.038 ^ab A^	1.060 ± 0.020 ^ab AB^	1.074 ^II^
Average	1.005 ^Z^	1.002 ^Z^	0.995 ^Z^	

Lowercase letters indicate the significance of differences for both variables, i.e., Cd dose and sorbent addition; uppercase letters indicate the significance of differences for sorbent type separately for each Cd dose; Roman numerals indicate the significance of differences for mean sorbent results, regardless of the cadmium dose; the letters Y, and Z indicate the significance of differences for the mean Cd dose results, regardless of sorbent addition.
